# Epidemic Cerebro-Spinal Meningitis

**Published:** 1864-08

**Authors:** G. A. Moses

**Affiliations:** Mobile, Alabama.


### CONFEDERATE STATES

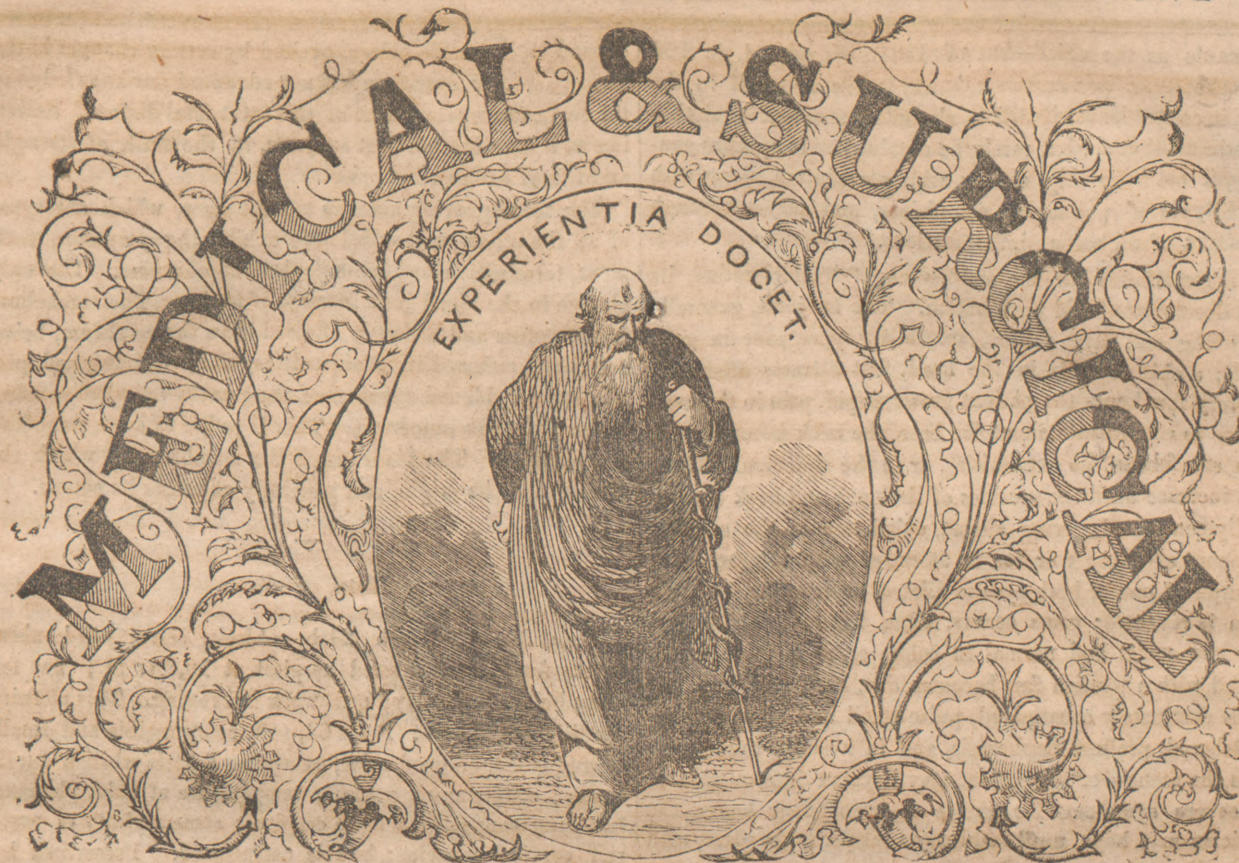

### JOURNAL.

VOL. I. RICHMOND, AUGUST, 1864. No. 8.

### ORIGINAL COMMUNICATIONS.

ART. I.—*Epidemic Cerebro-Spinal Meningitis.* By Surg'n G. A. MOSES, Mobile, Alabama.

During my connection with the army my attention has been at various times attracted by a disease which had hitherto never come under my observation. It is that type of cerebral disease, known as cerebro-spinal meningitis. It occurred, I believe, at Bowling Green, during the winter of 1861–'62. In the succeeding winter, while the army in the West was at Grenada, Miss., this disease made its appearance among the negroes employed upon the fortifications, and also among plantation hands in several adjoining counties. Again, during the past winter, I have observed it at this place, almost entirely confined to the blacks, both those employed by Government and others. Some cases have occurred among the citizens, principally in children.

The disease is marked by its rapid course, and fearfully frequent fatal termination. I have heard of but five *reported* recoveries, and have seen none.

* The first account I find, of a disease resembling this, but unnamed, dates in the year 1310, when it appeared in France; it is not again mentioned until 1503. A disease almost similar appeared in 1510 and 1517; after a very severe winter (1553) in Silesia, it carried off large numbers of the population. In 1580, associated, as now, with catarrhal affections, it killed no less than 10,000 persons in Rome, 12,000 in Madrid, and proportionately large numbers in other cities. During the civil wars in France, 1616, Ozenaur says, "The armies, both Catholic and Protestant, are decimated by a new disease," the subjects being attacked by "sudden and furious pain in the head." It lasted more than three months, and but few were saved. Sydenham reports it in 1661 and '64, as selecting the young and most robust subjects—as partaking of the type of typhus. In 1788, during an epidemic of typhus in Lyle, a disease appeared more nearly approaching to the character of the present epidemic. It was accompanied with tetanic convulsions and coma, the pia mater being chiefly involved. Not until 1830 was any name given this disease, when closer examination more fully indicated its seat and nature. Of late years it has occurred in many portions of the Confederacy. No sufficient cause has yet been assigned for its appearance or cessation.

M. Tourdes, in his valuable paper, has published statistics of attributable causes, and assigns the abuse of alcoholic

* Vide. History of Epidemic Cerebro-Spinal Meningitis. Bibliotique du Mèdecin Practicién. Vol. IX.

stimulants as the chief—but of 136 cases quoted, in 100 cases the cause is "*unknown*." As it has occurred in the army, it cannot be attributed to alcohol, as amongst its victims this stimulant could but rarely be obtained. It appears generally, if not invariably, during cold, wet winters, along with severe types of pulmonary complaints, and passes through the stages of all serous inflammations.

It is remarkable for the suddenness of its declaration, its rapid development and termination. The subjects, generally the young and most robust, are to all appearance in good health; a chill, or pain in the head, first attracts attention; in a very few hours the patient grows stupid, pain in the head appears to concentrate about the base, the neck becomes stiff, pains are felt in the extremities, or in the abdomen. These signs increase until the muscles of the neck and back become rapidly contracted, giving almost, opisthotonos; the smallest movement of a finger or toe is attended with intense pain; the pupil of one or both eyes is dilated or inactive, or their action is reversed; coma occurs, often trismus; the tongue, until now moist and normal in color, or, as is more usual, covered with a whitish fur, becomes dry, hard and swollen; bowels obstinately constipated, pulse small and slow, respiration labored, profuse diaphoresis, and in a short time death closes the scene—or the patient may have an intermission of the severe symptoms for twelve or twenty-four hours, the physician may hope, until suddenly a relapse takes place, with fatal ending.

Treatment has been of but little benefit—everything has been recommended and tried with poor success. *M. Rollet* advised general blood-letting, leeches and cups to length of spinal column, or actual cautery, with sinapisms and blisters of ammonia covering the whole body. Grissole, in addition, recommends mercury, Ganssauch trusts to quinia, Chauffard to opium in large doses. They all lost from sixty to eighty per cent. of the cases. After the disease has progressed to the extent it may in four or six hours, no medicine appears to act, croton oil failing to move the bowels to action.

The first symptom of the disease which attracts attention, appears generally to indicate not the commencement of disease, but its maturity, as in those cases which die in from ten to fifteen hours, with large effusion of lymph in the pia mater. Insidious in approach, it declares itself at a time when interference is of no avail.

I am indebted to Dr. S. C. Young, P. A. C. S., for information regarding the course of this disease at Grenada, Miss. Of thirty-five cases which came under his observation, he *knows* of no recovery; one was apparently improving, when, at the end of the third week, he was taken from the hospital. Some of the cases under his charge lived twelve and fifteen days, even longer. He thinks mercurials and stimulants promise the most success. Theory would seem to approve his opinion, but the great difficulty is to bring the patient under the influence of the remedy. The disease, as it has appeared in hospital here, runs its course with more uniform rapidity than it has before done, no case having lived through the fifth day. Like cholera and yellow fever, this epidemic appears to depend on a specific poison, excited by certain changes in the atmosphere. Experience has not advanced our knowledge of the real cause or treatment of this most fatal disease. Selecting as it does the hardiest subjects, in the flush of strength and life, it baffles all our skill.

The accompanying notes in several cases, which are types of all the others, will sufficiently exhibit the symptoms, treatment, termination and pathological appearances. The only change in the blood is an increase of fibrine. The cerebellum is more often and seriously affected than the cerebrum, being sometimes softened in its superficies, while the internal portion has a reddened appearance, the puncta vasculora seeming larger and more numerous. This I judge to be a secondary complication. The pia mater is the membrane in which the disease finds its origin, and generally exhausts its course.

---

*Notes of Cases.*

*Case No.* 1, March 24th.—Aleck, slave, aged about twenty-five years, entered hospital at 10, A. M.; has felt unwell since yesterday; quit work in evening. This morning condition as follows: Pulse 64, soft, compressible and small; tongue moist, of good color, excepting a little whitish fur in centre; countenance, natural; temperature of skin, pleasant; left lung congested, a little crepitus; some rigidity of posterior cervical muscles; bowels constipated. Prescribed hydrarg. c. mit.; pulv. jalapae aa. 10 gr.; wet cup chest; cold douche to head, to be applied continuously for half an hour, and intermitted for same length of time, and so alternately during day; whiskey, 1 drachm every half hour as long as necessary to produce effect. 5 P. M.—Pulse 80, very irregular, soft and quick; respiration 28; has been noisily delirious, and difficult to restrain in bed, since 1 P. M.; pupils largely dilated and inactive; passing urine involuntarily; spit out purgative this morning; skin of natural heat; cries, as though suffering. Continued whiskey and douche. March 25th, 9 A. M.—Pulse 94, small, soft and regular; respiration quiet and easy, at 28; pupils contracted to very small size and inactive; deep coma; left side of face warm, right side cool. Body and extremities warm; diaphoresis; tongue dry. Continued whiskey as often as could be administered. 4 P. M.—Pulse 110, fuller and soft; left pupil acting a little less promptly than normally; right pupil still contracted and motionless; face and skin same as this morning—not so much perspiration. Continued whiskey. 6 P. M.—No change, except that respiration is somewhat quicker. March 26—Died at 6½, A. M.; autopsy at 4, P. M.; body still unusually warm, although weather is cold; dura mater healthy; upon taking this off, the arachnoid is seen transparent, except when underlaid by lymph deposit; blood-vessels much congested and very tortuous; each vessel carries in its track lymph, more anteriorly than posteriorly; base of brain a mass of lymph, with some pus about the optic commissure across and around the pons varolii and medulla oblongata: arachnoid in some places bound down by bands of lymph; the deposit enters the convolutions, along with pia mater; lateral ventricles contain turbid serum, with flakes of lymph; spinal cord posteriorly, covered with same lymphy deposit.

*Case No.* 2, March 10th.—Henry, slave, age 25, entered hospital, March 9th, 9, P. M. Complained from the first of pain in the head, so intense that he constantly emitted cries and groans; pulse weak, at about 90; tongue moist and white; bowels active; pupils slow to act; had a chill before entering hospital. March 11th.—Pain increased; no change otherwise; is taking 5 grains iodide potash every hour. 12, A. M.—So noisy that he was moved to a detached room; seems to be suffering intensely; still conscious, but no answers can be elicited. 4, P. M.—Pulse somewhat increased in frequency; tongue dry; pupils dilated and almost inactive; coma commencing. 13th, 10, A. M.—Completely comatose; skin moist and cool; perfectly quiet; lying on the back for the first time; muscles of neck rather stiff; scalp blistered last night and stimulants administered; pulse slow and feeble. March 14th—Died at 4, A. M.: autopsy at 10; usual appearance of congestion and lymphy deposit between arachnoid and pia mater; pacchionian bodies enlarged; large deposit of lymph and pus at base of brain, especially over pons varolii and medulla oblongata; substance of cerebellum reddened and slightly softened superficially.

*Case No.* 3, March 19th, 5, P. M.—John, slave, age 28, has been sick for two days, before entering hospital, with diarrhœa; operations large and frequent; has taken three grains of opium. March 20th, 9 A. M.—Bowels quiet, and moved since last evening; pulse 148, small and soft; temperature of skin natural; inclined to be stupid; tongue dry and covered in centre with a white fur; pupils much contracted and motionless; complains of pain in head and neck, and in extremities, especially in superior; ordered whiskey, ½ ounce, every hour. Died at half-past four this afternoon: autopsy ——; dura mater, in several places near pacchionian bodies, adhered to subjacent membrane, so that in uncovering brain, portions of it were separated, leaving small, smooth irregular cavities, as of a slough; lymphy deposit along course of blood-vessels, and posterior to optic commissure; no disease in spinal chord.

*Case No.* 4.—Reported by Assistant-Surgeon J. H. Purifoy, entered February 24th, with some symptoms of pneumonia, which endured for a day before signs of meningitis occurred, when the case took all the usual course, of the above mentioned cases, with one marked peculiarity. The pupils after being dilated, became much contracted, and *expanded upon the admission of light.* Death occurred on the fifth day. Post mortem examination revealed: enlargement and an appearance of red hepatization of the pituitary body, in addition to the usual deposition of lymph, with some pus. This case was treated throughout with quinine and stimulants, after bleeding by cups to the extent of ten ounces.

